# Cost-effectiveness of Paxlovid in reducing severe COVID-19 and mortality in China

**DOI:** 10.3389/fpubh.2023.1174879

**Published:** 2023-06-19

**Authors:** Weina Zhang, Lanfang Li, Zhen Zhou, Qiao Liu, Guan Wang, Dan Liu

**Affiliations:** ^1^Department of Pharmacy, Northwest Women’s and Children’s Hospital, Xi’an, Shaanxi, China; ^2^Department of Pharmacy, Affiliated Hospital of Jining Medical University, Jining, China; ^3^School of Public Health and Preventive Medicine, Monash University, Melbourne, VIC, Australia; ^4^Department of Pharmacy, The Second Xiangya Hospital of Central South University, Changsha, China; ^5^State-Owned Assets Management Department, Northwest University of Political Science and Law, Xi’an, Shaanxi, China; ^6^Reproductive Medicine Center, Department of Obstetrics and Gynecology, The Second Xiangya Hospital of Central South University, Changsha, China

**Keywords:** COVID-19, Paxlovid, cost-effectiveness, affordable price, China

## Abstract

**Objectives:**

To assess the cost-effectiveness of Paxlovid in reducing severe COVID-19 and its associated morality, and to investigate the affordable price of Paxlovid in China.

**Materials and methods:**

Using a Markov model, two interventions by Paxlovid prescription (with and without prescription) were compared in terms of COVID-19-related clinical outcomes and economic loss. COVID-related costs were collected from the societal perspective. Effectiveness data were obtained from literature. The primary outcomes were total social cost, disability adjusted life-years (DALYs) and net monetary benefit (NMB). Scenario analyses were performed to investigate the affordable price of Paxlovid in China. Deterministic sensitivity analyses (DSA) and probabilistic sensitivity analysis (PSA) were performed to verify the model robustness.

**Results:**

Compared with the non-Paxlovid cohort, the NMBs of the Paxlovid cohort were only higher in the subgroup of patients aged over 80 years old, regardless of their vaccination status. Our scenario analysis found that, the price ceiling of Paxlovid/box for it to be cost-effective was RMB 8,993 (8,970–9,009) in those aged over 80 years old who were not vaccinated, which is the highest; and was RMB 35 (27–45) in those aged 40–59 years old who were vaccinated, which is the lowest. Sensitivity analyses found that the incremental NMB for the vaccinated people aged over 80 years was most sensitive to the efficacy of Paxlovid and the cost-effectiveness probability of Paxlovid increased with its decreasing price.

**Conclusion:**

Under the current marketing price of Paxlovid/box (RMB 1,890), using Paxlovid was only cost-effective in people aged over 80 years old regardless of their vaccination status.

## 1. Introduction

Since the outbreak of coronavirus disease 2019 (COVID-19) in China in December 2019, China has withstood multiple rounds of outbreaks from this highly contagious virus through continuous dynamic optimization and adjustment of its prevention and control measures (1–3). China’s anti-COVID-19 policies have successfully warded off a nationwide transmission of relatively strong pathogenic original strains and Delta variant. As a consequence, the direct COVID-19 disease burden, in particular, the number of deaths was largely reduced, and it has bought the time for the domestic medical institutions and pharmaceuticals to develop COVID-19 vaccines and antiviral drugs (4, 5). At the end of 2021, the Omicron variant and its sublineages, which became less virulent compared to previous variants, swiftly surpassed other variants to become the dominant lineages worldwide (6, 7). In a study analyzing data of 626,811 Chinese who were infected with Omicron BA.2 from Feb 2022 to June 2022 in Shanghai, most of whom were vaccinated, the overall asymptomatic infection rate was estimated to be 90.7% (95% confidence interval: 90.7–90.8%) (8).

In view of the evolution trend of COVID-19 to low virulence, the great progress on antiviral drugs and the high vaccination rate among Chinese population (9, 10), the China’s State Council joint COVID-19 Prevention and Control Mechanism downgraded the management of COVID-19 from Class A to Class B on December 26, 2022 (11). With the release of new COVID-19 treatment guideline on Jan 6, 2023 and the 10th version of the guideline on COVID-19 management on Jan 7, 2023 (12, 13), the priority for Chinese government to manage COVID-19 has now shifted from preventing COVID-19 infections towards preventing severe diseases from infections. This will inevitably lead to a dramatic rise in Omicron infections in the near future in view of China’s 1.4 billion population base and the high transmissibility of Omicron. There is an urgent need for Chinese government to find effective and affordable anti-COVID-19 drugs to respond to the upcoming major public health emergency. Nirmatrelvir-ritonavir (Paxlovid), an oral antiviral drug produced by Pfizer, have been proved that is highly effective in reducing severe and fatal COVID-19-associated outcomes in several population-based cohort studies (14, 15). Paxlovid was approved by the National Medical Products Administration (NMPA) to treat adults with mild-to-moderate COVID-19 infection who have a high risk for progression to severe illness (16). In view of the clear clinical benefit of Paxlovid, the Chinese government is looking forward to having it covered by the national medical insurance scheme to improve its accessibility, and the price negotiation with Pfizer was held on January 8, 2023. Unfortunately, due to its high quotation, the negotiation failed and Paxlovid was therefore not included in the National Reimbursement Drug List (NRDL) (17). Although the national medical insurance program will continue to reimburse patients for the use of Paxlovid until the end of March (18), its huge potential beneficiaries and the price of Chinese dollar of Renminbi (RMB) 1,890 per box will undoubtedly impose a substantial financial burden to the Chinese government.

An appropriate quotation from the Pfizer for the Paxlovid that conforms to China’s national conditions is the key for Paxlovid to be successfully included in the NRDL. To inform this we performed this analysis to assess the cost-effectiveness of Paxlovid in reducing severe COVID-19 and morality and to investigate the affordable price of Paxlovid for eligible population in China. Evidence generated from this study will be useful to inform the China’s National Healthcare Security Administration (NHSA) about the value of Paxlovid and has a potential in guiding and facilitating future price negotiations.

## 2. Methods

### 2.1. Overview

**Figure 1:**
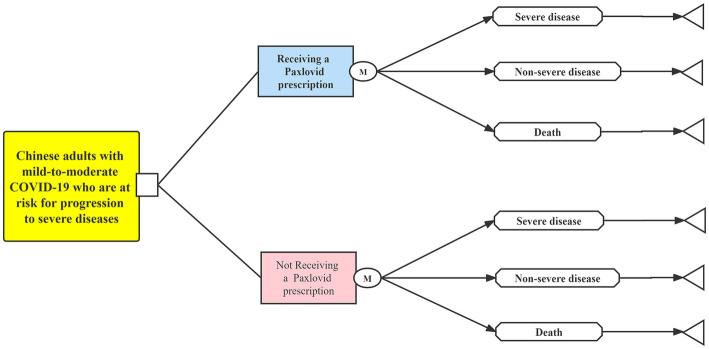
Markov model diagram. COVID-19, coronavirus disease 2019.

Using treeAge Pro Healthcare software (version 2021, https://www.treeage.com/), we built a Markov model to implement this cost-effectiveness analysis (Figure 1). This study was reported following the Consolidated Health Economic Evaluation Reporting Standards (19). Since only the existing data from published literature were used, our study was deemed exempt from the approval of the Chinese Ethics Review Committee (20).

### 2.2. Population and interventions

To evaluate the role of Paxlovid in reducing severe and fatal COVID-19 disease, two interventions by Paxlovid prescription (with and without prescription) were compared in terms of Omicron associated clinical outcomes and economic loss. Two hypothetical cohorts of 10 million adults with mild-to-moderate COVID-19 at a high risk for progression to severe illness were constructed for each intervention. The epidemiological data of the cohort with a prescription for Paxlovid (shortened as the Paxlovid cohort hereafter) was extracted from a real-world data study (8), which included all COVID-19 infections in Shanghai, China from February 26 to June 30, 2022 when Omicron variants predominated in China and Paxlovid was in sufficient supply. In view of China’s official lifting on all COVID-19 restrictions on January 8, 2023, the real-world Omicron epidemic data for the patient group who were eligible to but not prescribed Paxlovid (shortened as the non-Paxlovid cohort hereafter) was not available until the end of the study. Therefore, the reported hazard ratios (HRs) for Paxlovid prescription recipients vs. nonrecipients in terms of severe COVID-19 or mortality were used to estimate the relevant epidemic data for the non-Paxlovid cohort (14). Supplementary Table S1 detailed the epidemic data used for the studied population.

### 2.3. Effectiveness

This study used disability adjusted life-years (DALYs) to quantify disease burden for two hypothetical cohorts. DALY is a composite measure of disease burden, which was calculated as the sum of the years of life losts (YLLs) due to premature death and years lost due to disability (YLDs) (21).

YLLs were calculated using the following formula: $YLLs=N_{fatal}\times Y_{loss}$ , where *N_fatal_* is the number of deaths caused by Omicron infection, *Y_loss_* represents the loss of life expectancy at the age of death. YLDs were calculated for each non-fatal health state by multiplying the number of individuals who enter the state by the average duration of the state and disability weight (reflecting disease severity, scale of 0 to 1 with 0 denoting no disability). This study used a disability weight of 0.53 and a duration of 28 days for severe illness, and a disability weight of 0.17 and a duration of 14 days for non-severe illness (21). The parameters used for effectiveness estimation are listed in Supplementary Table S2.

### 2.4. Costs

We collected data of Omicron-related costs from a societal perspective, including Paxlovid costs, medical costs for severe and non-severe diseases; productivity losses due to COVID-19; and salaries paid to health workers. In this study, all costs were inflated to 2022 prices using domestic inflation rates derived from the China’s healthcare consumer price index in 2022 (22).

The recommended dosage of Paxlovid is the dosage of one box, that is, 300 mg (150 mg × 2 tablets) of nematrivir combined with 100 mg (100 mg × l tablets) of ritonavir, administered orally every 12 h for 5 consecutive days. The latest bid-winning price of 1,890 RMB for Paxlovid was retrieved from the national big-data platform (23). Individual-level medical cost (*M_c_*) for severe and non-severe diseases were retrieved from a cost-of-illness study conducted by Jin et al. (24). The total M*_c_* of each intervention was calculated as: $M_{c}=M_{c\left(severe\right)}\times N_{severe}+M_{c\left(non-severe\right)}\times N_{non-severe}$ , where *N_severe_* and *N_non-severe_* represent the number of severe and non-severe COVID-19 cases, respectively.

To estimate COVID-19 induced-productivity losses (*PL_c_*) for each intervention, the human capital approach was used as follows: $PL_{c}=S_{daily}\times R_{employment}\times N_{severe}\times D_{h}$ (24), where *S_daily_* represents the average daily salaries of Chinese employees in 2022; *R_employment_* represents the employment rate, which varied by age; *D_h_* is the reported average days of productivity losses for COVID-19 diseases (14 day for non-severe diseases and 28 days for severe diseases) (24). The average daily salary of Chinese employees was estimated to be RMB 293 based on an annual income of RMB 106,837 (25). The employment rate for adults aged 18–59 years old was set as 94.4% and for adults over 60 as 0% (25).

This model only considered the salaries paid to health workers working in areas related to the treatment of severe COVID-19, because most persons with non-severe infections were self-treated at home according to the guidance of the latest COVID-19 Homecare Guidelines (26). When estimating the salaries paid to health workers (*S_c_*), we followed the following steps. ***Step 1***, we determined the ratio between hospital beds and health workers for managing severe COVID-19 in China. According to the national statistics (25), the hospital beds and health workers in the intensive care unit should be allocated in a ratio of about 1:4. ***Step 2,*** we calculated the number of health workers required for severe COVID-19 as the number of severe COVID-19 cases multiplied by the ratio between health workers and hospital beds. ***Step 3,*** the formula: $S_{total}=S_{daily}\times N_{staffs}\times D_{s}$ was used to calculate the total salaries paid to health workers for each intervention, where *S_daily_* refers to the average daily salary of healthcare industry in China, *N_staff_* refers to the required number of health workers, *D_s_* refers to the working days that health workers work for treating each severe COVID-19 case, which is equal to the average hospitalization days for patients with severe COVID-19 in this study. The average daily salary of personnel in healthcare industry in China was estimated to be RMB 347 based on an annual income of RMB 126,828 (25). Supplementary Table S3 summarizes the parameters related to cost estimation.

### 2.5. Cost-effectiveness analysis

This study used net monetary benefit (NMB) to measure the relative cost-effectiveness between two interventions. NMB is a summary statistic that represents the net value of an intervention in monetary terms after considering costs (21), which was calculated as $NMB_{Intervertion}=DALY_{Intervertion}\times WTP-\cos t_{Intervertion}$ . This study used the China’s *per capita* gross domestic product in 2022 as the willingness-to pay (WTP) threshold for each averted DALY, which is RMB 85,698 according to the National Bureau of statistics (24). The intervention with a higher NMB was considered to be more cost-effective compare with the alternative.

### 2.6. Scenario analysis

We designed a what-if scenario, that is, if a Paxlovid prescription is cost-effective for Chinese adults with mild-to-moderate COVID-19 who are at a high risk for progression to severe illness, what the affordable price of Paxlovid would be in China.

### 2.7. Sensitivity analyses

Two sensitivity analyses were employed to verify the model robustness, including (1) deterministic sensitivity analyses (DSA) to identify the sensitive factors by varying model parameters individually and (2) probabilistic sensitivity analysis (PSA) to examine the impact of joint uncertainty of multiple parameters simultaneously. Each parameter fluctuated between the baseline value minus 25% and the baseline value plus 25% in the DSA and was matched an appropriate distribution in the PSA (Supplementary Tables S2, S3).

## 3. Results

### 3.1. NMB

For Chinese adults with mild-to-moderate COVID-19 who are at a risk for progression to severe diseases, compared with the non-Paxlovid cohort, the costs was higher in the Paxlovid cohort in all subgroups except for the group of patients aged over 80 years old in the context of the current market price of Paxlovid/box (RMB 1,890); in addition, using Paxlovid was associated with lower DALYs, except for the 18–39 years old patient subgroup who were vaccinated (Table 1). In general, the NMBs estimated for the Paxlovid cohort were only higher in the subgroup of patients aged over 80 old, regardless of their vaccination status.

**Table 1:** NMBs estimated for cohort with/without Paxlovid prescription.

Subgroups	Cohort	Cost (RMB in 10 millions)		DALY		NMB (RMB in 10 millions)	Cost-effectiveness intervention
		Total	Difference	Total	Difference		
Vaccinated_18–39 years old	Non-Paxlovid	13,385		3,900		−13,352	√
	Paxlovid	15,275	1,890	3,900	0	−15,242	
Vaccinated_40–59 years old	Non-Paxlovid	13,459		4,149		−13,424	√
	Paxlovid	15,312	1,853	4,024	−124	−15,278	
Vaccinated_60–79 years old	Non-Paxlovid	9,895		5,227		−9,850	√
	Paxlovid	11,594	1,699	4,564	−664	−11,555	
Vaccinated_ > 80 years old	Non-Paxlovid	13,593		18,086		−13,438	
	Paxlovid	13,443	−150	10,993	−7,093	−13,349	√
Unvaccinated_18–39 years old	Non-Paxlovid	13,508		4,315		−13,471	√
	Paxlovid	15,337	1,828	4,107	−207	−15,302	
Unvaccinated_40–59 years old	Non-Paxlovid	14,247		6,804		−14,189	√
	Paxlovid	15,706	1,459	5,352	−1,452	−15,661	
Unvaccinated_60–79 years old	Non-Paxlovid	12,972		15,929		−12,836	√
	Paxlovid	13,133	160	9,915	−6,015	−13,048	
Unvaccinated_ > 80 years old	Non-Paxlovid	28,051		68,360		−27,465	
	Paxlovid	20,672	−7,379	36,130	−32,230	−20,362	√

RMB, Renminbi; DALY, disability adjusted life-years; NMB, net monetary benefit.

### 3.2. Scenario analysis

Scenario analysis results are summarized in Table 2. Supplementary Figure S1 shows the relationship between the price of Paxlovid/box and the incremental NMB of Paxlovid versus non-Paxlovid. For the 18–39 years old who were vaccinated, even reducing the price of Paxlovid/ box to RMB 0 could not make the incremental NMB between the Paxlovid cohort and the non-Paxlovid cohort positive. The price ceiling of Paxlovid/box for it to be cost-effective was RMB 8,993 (RMB 8,970-9,009) in those aged over 80 years old who were vaccinated, which is the highest; and was RMB 35 (RMB 27–45) in those aged 40–59 years old who were vaccinated, which is the lowest.

**Table 2:** The price ceiling of Paxlovid/box investigated for subgroups.

Subgroups	Affordable price ceiling of Paxlovid/box for Paxlovid to be cost-effective (95% CI), RMB
vaccinated_18–39 years old	/
vaccinated_40–59 years old	35 (27–45)
vaccinated_60–79 years old	185 (171–200)
vaccinated_ > 80 years old	1,979 (1,964–1,994)
unvaccinated_18–39 years old	59 (42–76)
unvaccinated_40–59 years old	418 (401–435)
unvaccinated_60–79 years old	1,678 (1,663–1,693)
unvaccinated_ > 80 years old	8,993 (8,970–9,009)

CI, confidence interval; RMB, Renminbi.

### 3.3. Sensitivity analyses

Supplementary Figure S2 showed the results of deterministic sensitivity analysis for the vaccinated subgroups who were aged over 80. The incremental NMB was most sensitive to the HR of severe COVID-19 and mortality with Paxlovid use. For instance, decreasing the HR from 0.50 to 0.38 increased the incremental NMB of Paxlovid vs. non-Paxlovid to RMB 1,216, whereas increasing the HR to 0.63 decreased the incremental NMB to RMB -587. Other model parameters with the potential to decrease the incremental NMB to below zero included the market price of Paxlovid/box, the risk of severe diseases upon infection (ISR) estimated for Paxlovid prescription recipients and the medical costs for severe COVID-19.

Supplementary Figure S3 illustrated the cost-effectiveness probability of Paxlovid versus non-Paxlovid under the circumstances of different marketing prices of Paxlovid/box. In general, the probability increased with the decreasing price of Paxlovid /box in different subgroups.

## 4. Discussion

On the basis of a Markov model, we concluded that at the current bid-winning price of RMB 1890 of Paxlovid per box, prescribing Paxlovid to Chinese adults with mild-to-moderate COVID-19 who are at a risk for progression to severe diseases was cost-effective only in patients aged over 80, regardless of their vaccination status. Scenario analysis aimed at investigating the affordable price of Paxlovid in eligible adults with COVID-19 revealed that for the 18–39 years old who were vaccinated, even making Paxlovid free could not make Paxlovid to be more cost-effective compared with no use. The reason for this observation is that the zero ISR of this subgroup leads to the failure of Paxlovid to show an effect in reducing severe diseases and mortality. Moreover, under the WTP threshold of RMB 85,698 used in the analysis, the affordable price ceilings of Paxlovid/box for Paxlovid to be cost-effective were generally higher in the unvaccinated population than in the vaccinated population, and the price ceiling elevated with increasing age. This is mainly because the protective effect of Paxlovid on reducing severe COVID-19-associated outcomes was stronger among the population at a higher risk for progression to severe diseases, such as the older adults or those who were not vaccinated (14, 15).

In the DSA, we only evaluated the impact of the uncertainty around model parameters on the incremental NMB of the subgroup of patients aged over 80 years old who were vaccinated. There are two reasons for this practice: first, according to the latest data issued by the China’s State Council joint COVID-19 Prevention and Control Mechanism, the vaccination coverage rate in China has reached 92.9% (27); second, a China-based real-world study that included 612,597 Omicron infection cases reported a median age of 83 years of patients who had developed severe illness from COVID-19 and a median age of 86 years of patients who died for COVID-19. The DSA results indicated that a weaker effect of Paxlovid in reducing severe COVID-19 and mortality, a higher market price of Paxlovid in China, a lower risk of severe diseases in this subgroup, and a lower medical cost of severe diseases (or in other words, less severe diseases) would make Paxlovid use less cost-effective than no use. Among the four most influential parameters, the market price of Paxlovid is currently the only one that can be changed through China’s policy interventions. It is interesting to note that, as suggested by our DSA results, if SARS-CoV-2 evolves towards less virulent as expected, the clinical value of paxlovid would be largely compromised in the future.

Our PSA explored the cost-effectiveness probability of Paxlovid prescription versus no prescription among vaccinated subgroups with different ages. An overall trend of an increasing cost-effectiveness probability of the Paxlovid cohort was observed with the price of Paxlovid/box decreased. Therefore, the only way to make the cost of Paxlovid commensurate with its clinical value would be to lower its price. Synthesizing our scenario analysis results, we found that a 90% reduction in the price per box of Paxlovid would render the Paxlovid cohort to become cost-effective in the subgroup of vaccinated patients aged 60–79 years old. As such, a 98% reduction in the price per box of Paxlovid would render the Paxlovid to become cost-effective in the subgroup of vaccinated patients aged 40–59 years old. Although NHSA negotiation with pharmaceuticals is among the most effective measures to reduce drug prices in China, a drug price reduction by more than 90% is almost impossible according to historical NHSA’s negotiation results in recent years (28–30). Therefore, we can conservatively predict that use of Palxovid in eligible COVID-19 adults aged 40–79 would not be considered cost-effective.

To our knowledge, this is the first study that assessed the cost-effectiveness of Paxlovid in reducing severe COVID-19 and morality among Chinese adults with mild-to-moderate COVID-19 who are at a risk for progression to severe diseases under the current ongoing Omicron waves. Moreover, this study is also the first to investigate the affordable price of Paxlovid for eligible adults at different ages. The findings of our analysis contribute important evidence to support the use of Paxlovid at the current bid-winning price of RMB 1890 per box as a cost-effective treatment for eligible adults over 80 years old, regardless of their vaccination status; however, it does not support the use of Paxlovid at any bid-winning price (even if Paxlovid is free) as a cost-effective treatment for eligible vaccinated adults aged 18–39 years old. For eligible vaccinated adults aged 40–79 years old, a more than 90% of Paxlovid’s price reduction would make the Paxlovid cost-effective in the current wave of Omicron variants.

This study has several limitations. First, epidemic data for the eligible adults who were not prescribed Paxlovid were not available from the real world. More authoritative data are needed to validate our results. Second, the long-term impacts of COVID-19 on health, such as the cancelation or delay of the regular monitoring and care for persons with chronic conditions (e.g., cancer, diabetes, cardiovascular diseases, chronic liver disease) (31), as well as the delay of outpatient visits were not captured. Third, although we have tried our best to collect Omicron-related costs from the societal perspective, some cost components were not counted in the model, such as the the cost for use of any over-the-counter medicines. Fourth, due to the lack of head-to-head clinical data, this model did not compare the use of Paxlovid with a domestic anti-COVID-19 drug, such as Azhidine.

## 5. Conclusion

For Chinese adults with mild-to-moderate COVID-19 who are at a risk for progression to severe diseases, under the current market price of Paxlovid/box (RMB 1,890), using Paxlovid was only cost-effective in patients aged over 80 old regardless of their vaccination status.

## Data availability statement

The original contributions presented in the study are included in the article/Supplementary material, further inquiries can be directed to the corresponding authors.

## Author contributions

GW and DL had full access to all of the data in the study and took responsibility for the integrity of the data and the accuracy of the data analysis and statistical analysis. WZ, LL, ZZ, QL, GW, and DL concept and design, acquisition, analysis, interpretation of data, and critical revision of the manuscript for important intellectual content. WZ and ZZ drafting of the manuscript. All authors contributed to the article and approved the submitted version.

## Conflict of interest

The authors declare that the research was conducted in the absence of any commercial or financial relationships that could be construed as a potential conflict of interest.

## Publisher’s note

All claims expressed in this article are solely those of the authors and do not necessarily represent those of their affiliated organizations, or those of the publisher, the editors and the reviewers. Any product that may be evaluated in this article, or claim that may be made by its manufacturer, is not guaranteed or endorsed by the publisher.
