# Supplementary material for: Cost-effectiveness of Paxlovid in reducing severe COVID-19 and mortality in China
Source: Front Public Health. 2023 Jun 19;11:1174879. doi: 10.3389/fpubh.2023.1174879 (PMC10315619; doi:10.3389/fpubh.2023.1174879)
Supplement: Supplementary file 1 [file Data_Sheet_1.docx]

**Supplementary File**

**Table S1** The epidemic data of the studied population.

**Table S2** Parameters related to effectiveness estimations.

**Table S3**: Parameters related to cost estimations

**Figure S1** The relationship between the price of Paxlovid/box and the incremental NMB of Paxlovid versus non-Paxlovid. NMB, net monetary benefit; RMB, Renminbi; HR, hazard ratios.

**Figure S2** The results of deterministic sensitivity analysis for the previously vaccinated subgroups who aged over 80 years old. NMB, net monetary benefit; RMB, Renminbi; HR, hazard ratio; ISR, risk of severe diseases upon infection.

**Figure S3**. The cost-effectiveness probability of Paxlovid versus non-Paxlovid under different marketing prices of Paxlovid per box. RMB, Renminbi.

**Table S1 The epidemic data of the studied population**

| Age | Cohort with Paxlovid prescription | | | | Cohort without Paxlovid prescription | | | |
| --- | --- | --- | --- | --- | --- | --- | --- | --- |
|  | Adjusted IFR(%) | | Adjusted ISR(%) | | Adjusted IFR(%) | | Adjusted ISR(%) | |
|  | Vaccinated | Unvaccinated | Vaccinated | Unvaccinated | Vaccinated | Unvaccinated | Vaccinated | Unvaccinated |
| 18-39 years old | 0.00 | 0.01 | 0.00 | 0.04 | 0.00 | 0.02 | 0.00 | 0.08 |
| 40-59 years old | 0.00 | 0.07 | 0.03 | 0.28 | 0.00 | 0.14 | 0.06 | 0.56 |
| 60-79 years old | 0.02 | 0.31 | 0.14 | 1.14 | 0.04 | 0.62 | 0.28 | 2.28 |
| >80 years old | 0.25 | 2.22 | 1.46 | 5.55 | 0.50 | 4.44 | 2.92 | 11.10 |

*IFR, infection fatality ratio; ISR, risk of severe/critical disease upon infection.*

**The IFRs and ISRs of the cohort without Paxlovid prescription were calculated as the corresponding IFRs and ISRs of the cohort with Paxlovid prescription divided by the hazard ratios of 0.50.*

**Table S2 Parameters related to effectiveness estimations**

| **Parameters** | **Base case value** (Ranges) | **Distribution** | **Source** |
| --- | --- | --- | --- |
| Disability weight of mild/moderate diseases | 0.010 (0.008-0.0013） | Beta distribution (61,6024) | Zhao J,et al |
| Duration of mild/moderate diseases (year) | 0.039 (0.029-0.049) | Gamma distribution (16,410) | Zhao J,et al |
| Disability weight of severe/critical diseases | 0.530 (0.398-0.663) | Beta distribution (29,26) | Zhao J,et al |
| Duration of severe/critical diseases (year) | 0.079 (0.059-0.098) | Gamma distribution (16,203) | Zhao J,et al |

Table S3: Parameters related to cost estimations

| **Parameters** | **Base case value (Ranges)** | **Distribution** | **Source** |
| --- | --- | --- | --- |
| ***P****p* | 1,890 (1,418-2,363) | Gamma distribution (16, 0.0085) | National database |
| Mc |  |  |  |
| Mc(severe) (RMB) | 89,942 (67,457-112,428) | Gamma distribution (16, 0.0002) | Jin H, et al |
| Mc(non-severe) (RMB) | 9,513 (7,315-11,891) | Gamma distribution (16,0.0017) | Jin H, et al |
| **PLc** |  |  |  |
| Sdaily- Overall industry (RMB) | 293 (220-366) | Gamma distribution (16,0.0546) | National Bureau of Statistics |
| Remployment-18-59 years old | 94.4% (70.8%-100.0%) | Beta distribution (3,0.2042) | National Bureau of Statistics |
| Remployment->60 years old | 0 | / | National Bureau of Statistics |
| Dh(severe) | 28 (21-35) | Gamma distribution (16,0.5714) | Zhao J,et al |
| Dh(non-severe) | 14 (11-18) | Gamma distribution (16,1.1429) | Zhao J,et al |
| **SC** |  |  |  |
| Sdaily- Health industry (RMB) | 347 (261-434) | Gamma distribution (16,0.0461) | National Bureau of Statistics |
| *R_hospital beds vs health workers_* | 0.25 (0.19-0.31) | Beta distribution (46, 138) | National Bureau of Statistics |
| ISR(chort with Paxlovid prescription) |  |  |  |
| ISRvaccinated-18-39 years old | 0 | / | Chen X, et al |
| ISRvaccinated-40-59 years old | 0.03 (0.02-0.04) | Beta distribution (61,204762) | Chen X, et al |
| ISRvaccinated-60-79 years old | 0.14 (0.11-0.18) | Beta distribution (61,43781) | Chen X, et al |
| ISRvaccinated->80years old | 1.46 (1.10-1.83) | Beta distribution (61,4088) | Chen X, et al |
| ISRunvaccinated-18-39 years old | 0.04 (0.03-0.05) | Beta distribution (61,153541) | Chen X, et al |
| ISRunvaccinated-40-59 years old | 0.28 (0.21-0.35) | Beta distribution (61,21829) | Chen X, et al |
| ISRunvaccinated-60-79 years old | 1.14 (0.86-1.43) | Beta distribution (61,5269) | Chen X, et al |
| ISRunvaccinated->80 years old | 5.55 (4.16-6.94) | Beta distribution (58,988) | Chen X, et al |
| *HR_Paxlovid vs non-paxlovid_* | 0.50 (0.38-0.63) | Beta distribution (31, 31) | Shah MM,et al |

*H_c_, hospitalization cost; ISR, risk of severe/critical disease upon infection; PL_c_, productivity losses-related cost losses; S_daily_, average daily salaries;* Remployment, *employment rate;* Dh , *average hospitalization days; R_hospital beds vs health workers_*, ratio between hospital beds *and* health workers.

**Figure S1** The relationship between the price of Paxlovid/box and the incremental NMB of Paxlovid versus non-Paxlovid.


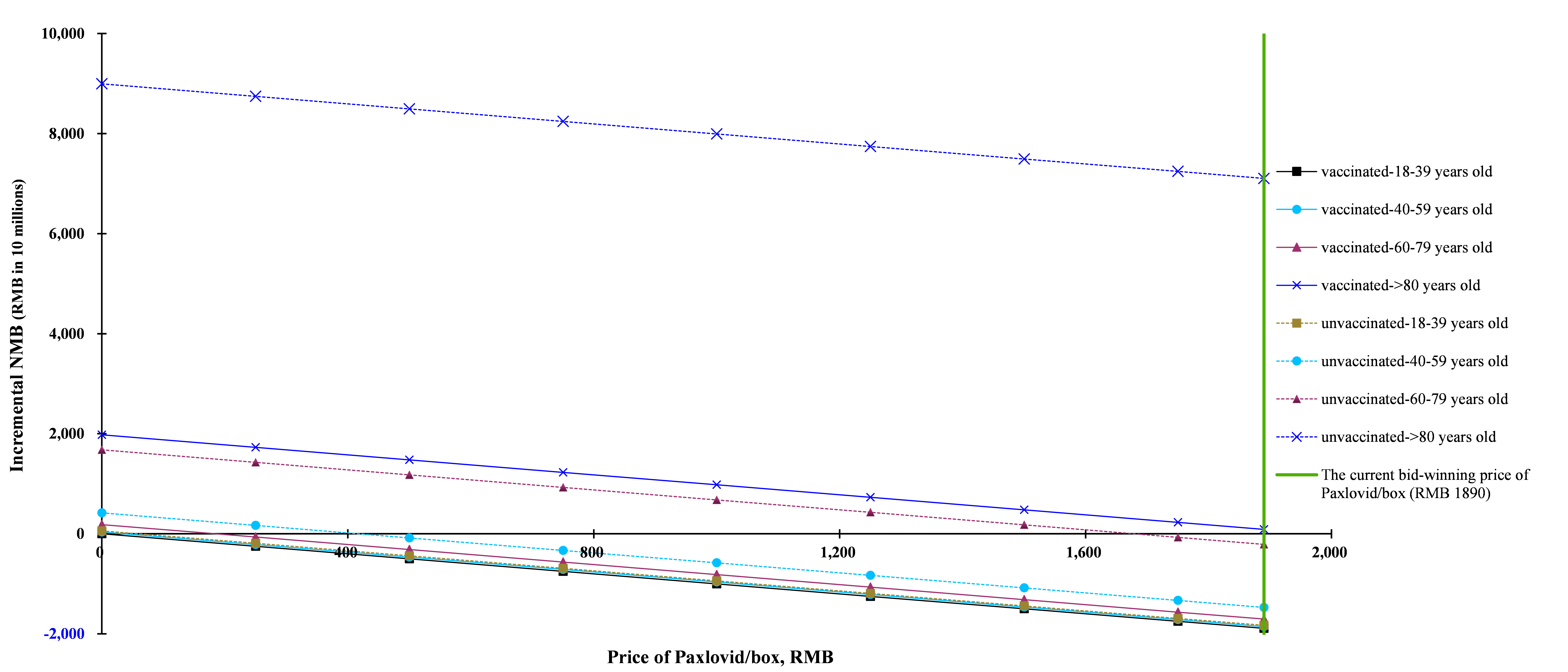


NMB, net monetary benefit; RMB, Renminbi; HR, hazard ratios.

**Figure S2** The results of deterministic sensitivity analysis for the previously vaccinated subgroups who aged over 80 years old.
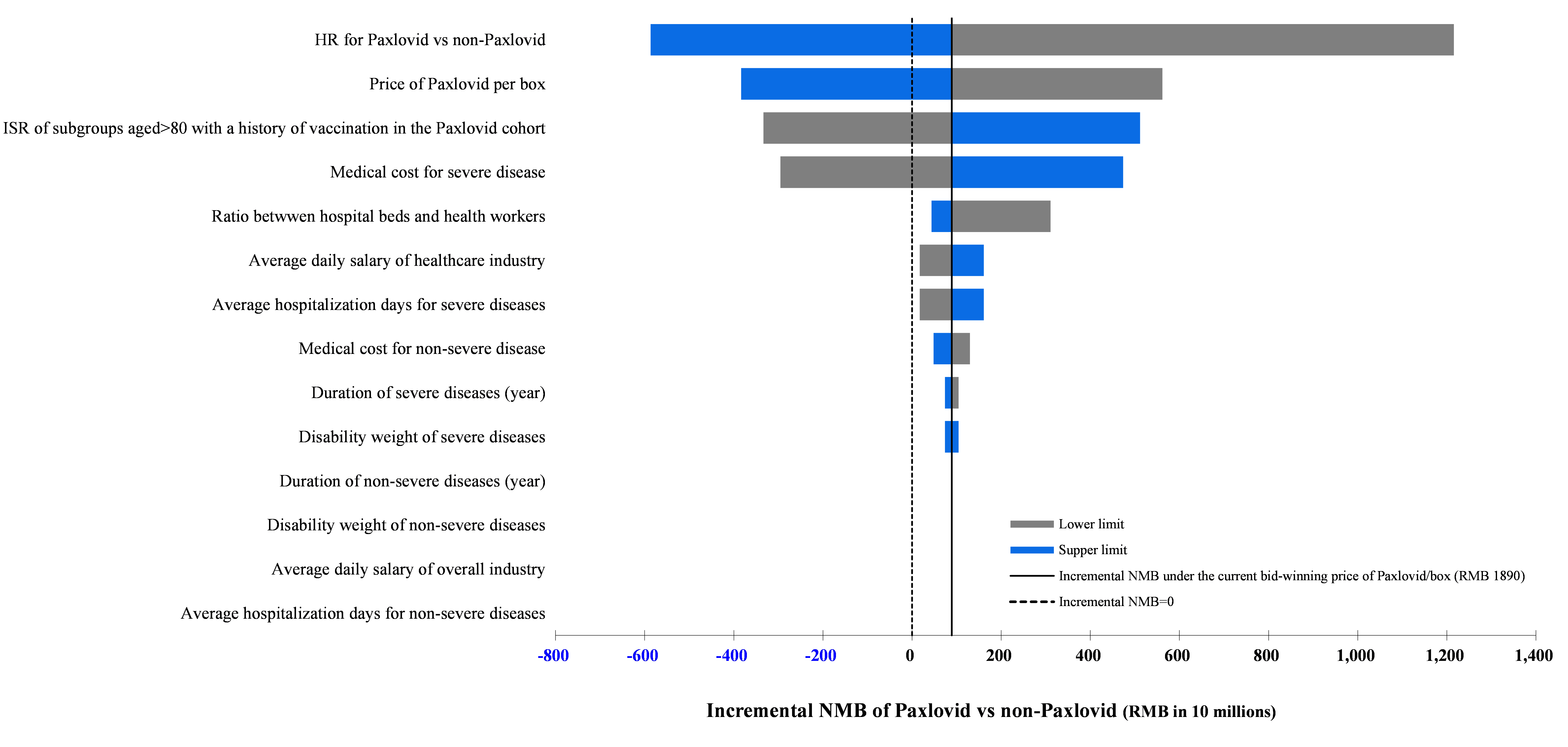


NMB, net monetary benefit; RMB, Renminbi; HR, hazard ratio; ISR, risk of severe diseases upon infection.

**Figure S3**. The cost-effectiveness probability of Paxlovid versus non-Paxlovid under different marketing prices of Paxlovid per box


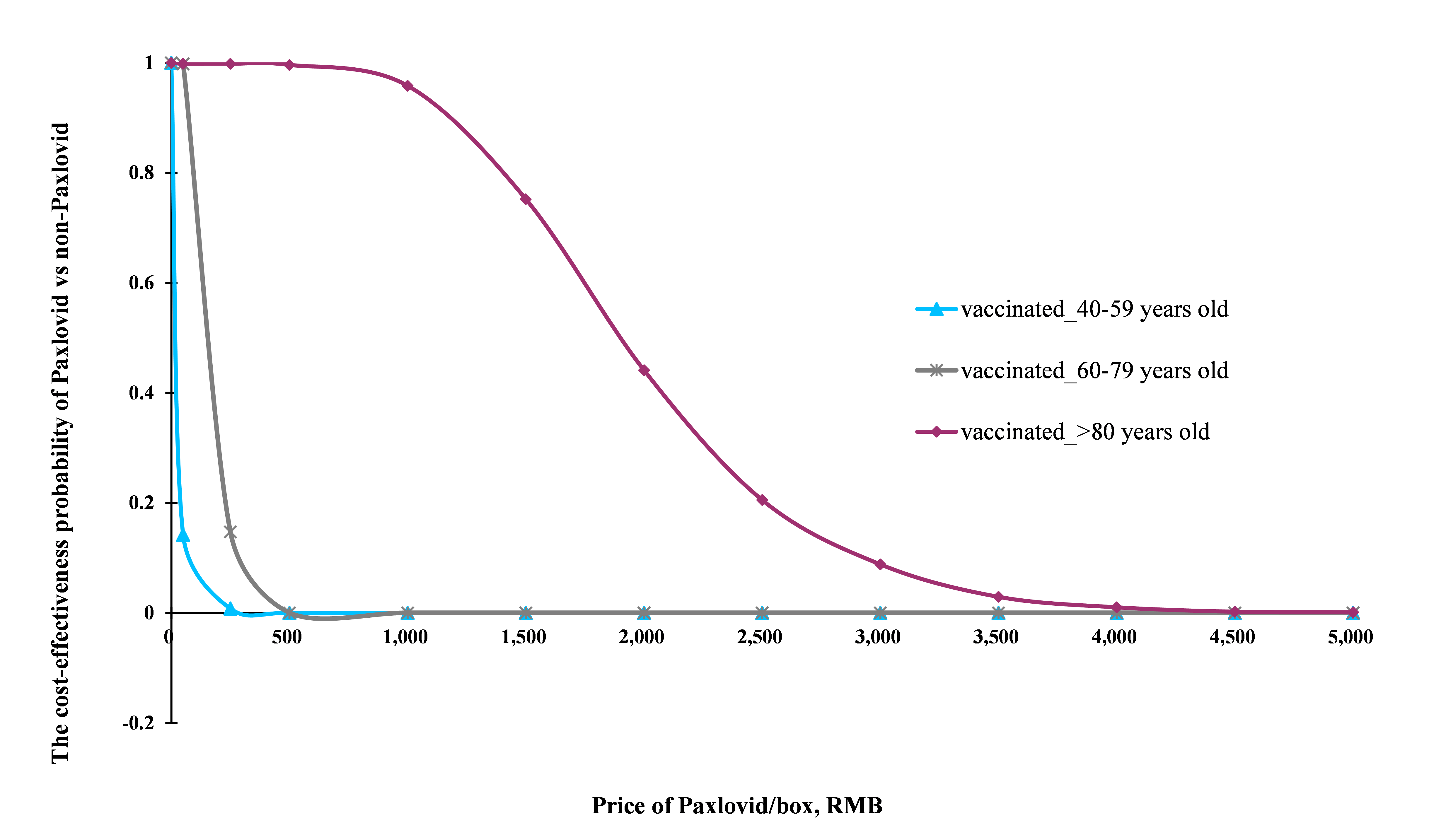


RMB, Renminbi.
